# PrEP: controversy, agency and ownership

**DOI:** 10.7448/IAS.19.7.21120

**Published:** 2016-10-18

**Authors:** Gus P Cairns, Kane Race, Pedro Goicochea

**Affiliations:** 1 European AIDS Treatment Group, Brussels, Belgium; 2 NAM Publications, London, UK; 3 Department of Gender and Cultural Studies, Faculty of Arts and Social Sciences, School of Philosophical and Historical Inquiry, University of Sydney, Sydney, NSW, Australia; 4 The Forum for Collaborative HIV Research, School of Public Health, University of California Berkeley, Berkeley, CA, USA

**Keywords:** HIV, HIV prevention, pre-exposure prophylaxis, sociology of HIV, HIV prevention implementation, key affected populations, men who have sex with men

## Abstract

Pre-exposure prophylaxis (PrEP) has been and continues to be an intervention that causes controversy and debate between stakeholders involved in providing or advocating for it, and within communities in need of it. These controversies extend beyond the intrinsically complex issues of making it available. In this commentary, some of the possible roots of the air of dissent and drama that accompanies PrEP are explored. The similarities between the controversies that dogged the earliest human trials of PrEP and the ones we see today in the era of licensing and implementation are explored. We outline five mediating principles or cultural norms that may influence arguments about PrEP differently. Three areas of specific concern are identified: medical risk versus benefit, distrust and fear of healthcare interventions, and fears for individual responsibility and community cohesion. The fear that PrEP may somehow represent a loss of control over one or more of these domains is suggested as an underlying factor. The development of countervailing measures, to institute greater community “ownership” of PrEP, and concomitant improvements in the sense of individual agency over sexual risk are outlined and recommended.

## Introduction

Although the efficacy of pre-exposure prophylaxis (PrEP) has been proven, the roll-out and general scale-up of PrEP face major challenges, including the potential for creating ongoing polarization in the field. The issues around the roll-out of PrEP have changed over time, but the controversy remains.

In this article, we look at the responses to the main controversies in the history of PrEP research and examine what social attitudes and cultural beliefs about health, illness and power may underpin them.

## The constituents of controversy

Lasting views and attitudes to PrEP emerged during the first clinical trials. They persist, somewhat transformed, among attitudes to the roll-out of PrEP today. Many are rationally based on concerns about PrEP or its impact. The concerns are potentially magnified by the idea that, in the era of the Internet, what previously might have been considered beliefs about health now is considered knowledge, even though that knowledge is partial [1]. Many other health beliefs based on personal, political or religious factors contribute to current perceptions of PrEP. These are often mediated by other cultural norms; factors include a tension between individualism and communitarianism in human responsibility, the role of colonialism in creating distrust towards former “oppressors,” religious perspectives and discourses about programmatic rationality (i.e. equity, cost–benefit ratio and sustainability). We argue that these factors generate three areas of debate discussed subsequently.

## Risk versus benefit

This involves a straightforward fear that PrEP will do more harm than good. In the early abandoned PrEP trials (e.g. the Cambodia or Cameroon), participants, their partners, relatives and peers developed beliefs that the pills contained HIV or that researchers were either deliberately infecting participants in blood tests or allowing them to be infected. Beliefs like these are different to the belief that PrEP will lead to toxicities. They are based on suspicions of malignant intent by researchers or the healthcare professionals, rather than on intrinsic medical and pharmaceutical risks. They have been both persistent and markedly more common in populations that are disadvantaged or socio-economically distanced from the researchers. Early trials were also associated with controversies linked to the perception that HIV care and treatment would not be available to people who seroconverted during the trial [2].

A further concern raised is that PrEP is doomed to ineffectiveness because of consequent increased condomless sex or “condom migration.” This is deduced from assumptions that even modest reductions of condom use will negate the effectiveness of PrEP. Contrary to this, a recently published mathematical model demonstrated that while PrEP may have an impact on rates of condom use, even zero condom use would not entirely abrogate the effectiveness of PrEP [3].

An associated concern is that more condomless sex due to PrEP will lead to more sexually transmitted infections (STIs). Again, evidence would show that while STIs have undoubtedly been increasing in men who have sex with men in recent years, the increase predates the introduction of PrEP. Another take is that PrEP is a response to historical decreases in condomless sex rather than a contributor to them.

Finally, controversy has occurred around PrEP access and whether drug would be made available post clinical trial [4]. More recently, attention has focused on what happens to PrEP users when after some time individual's risk changes – will PrEP eligibility and therefore access for that individual change?

## Autonomy and responsibility

Other concerns centre on the fear that PrEP could cause *social* harm and disempower key affected populations in ways that would cause long-term damage to their autonomy. Women's risk of HIV tends to be viewed as a function of their *vulnerability* to exploitation, violence and patriarchal moralities. But gay men's risk tends to be viewed as a function of their *responsibility* and therefore reducing that risk is their personal duty. In particular, PrEP is said to have the potential to erode gay men's sexual responsibility by enabling “unprotected” sex with multiple partners or, rather, reducing the anxiety that would formerly have prevented it.

On a more macro level, this social harm could reduce a community's power to demand structural and other resources that could be used to prevent HIV, rather than regulating outcomes of people's behaviour with medications.

## PrEP, control and agency

The list of the cultural concerns that have informed key strands of community opposition to PrEP in this article are incomplete. The issues we have not discussed include the idea that PrEP will “medicalize” what should properly be regarded as a structural and social issue and the fact that PrEP simply exemplifies global economic and health disparities an intervention that caters to affected communities in rich countries when over half the people with HIV in the world still cannot get treatment.

PrEP has been described by Professor Robert Grant of UCSF as a “demand-driven” intervention [5], meaning that “the indication for PrEP is that someone asks for it.” This implies that people are good at determining their own risk and that overly tight criteria for offering PrEP are unnecessary because people will self-regulate in terms of use and uptake. But it may also be taken to imply that PrEP will *only* work if it becomes part of a person's strategy for maintaining their health and quality of life. PrEP must not just be a medical prescription, but a tool to enable agency.

In the analysis of what went wrong in the early PrEP trials in Cameroon and Cambodia, USAID and the Global Campaign for Microbicides explain that while the trial participants were *consulted* about the trials, they were not *involved* in the planning. Involvement, the sense of actually being a stakeholder (whether in the design of clinical trials or in the construction of health strategies with one's healthcare provider), is a strong predictor of adherence, as qualitative studies of PrEP trials have proved [6].

Clinical researchers have increasingly sought to involve the communities in which PrEP is being studied in the actual design and running of trials. It is possible that one of the reasons the iPrEx trial was the first PrEP trial to report a successful result was due to a lengthy and intensive process of community consultation that occurred before and throughout the trial. This community consultation was not just an exercise in social research but also a way of actively preparing the varied MSM communities involved to develop a sense of being stakeholders [7].

Given the highly controversial nature of PrEP, and the involvement of ACT-UP Paris in activism against the Cambodia and Cameroon PrEP trials, the coordinators of the Ipergay trial actively sought to involve the relevant communities in trial design and delivery, including the counselling and peer-support aspects of the research.

In the PROUD trial, there was also an active community engagement group and a community representative (the lead author of this piece) served as the trial's co-chair. Increased community involvement has paid off, even in trials among the more difficult and disenfranchised populations. Although the intervention was found to be ineffective, the FACTS001 trial of a tenofovir gel microbicide was not marked by the same distrust as was seen in VOICE and Fem-PrEP; participants’ actual adherence matched their reported adherence, and the intervention did not work because it did not fit with existing social practices [8].

## Conclusions

PrEP is on the verge of wide adoption in several countries around the world, and the US experience suggests it should be considered as a public health measure. In Europe, North America, Africa and certain other countries such as Australia and Thailand, PrEP is being implemented in a piecemeal manner amidst the ongoing debate this intervention has generated. Its adoption has been facilitated by activist groups in the United States (see www.facebook.com/groups/PrEPFacts), the UK (see www.prepster.info and www.iwantprepnow.co.uk), France (see www.facebook.com/groups/PrepDial) and a number of others using various platforms including social media. In countries where PrEP is not yet available, affected communities are self-organizing to buy generic PrEP online, and there has been an upsurge of interest in and awareness of this intervention.

There continue to be setbacks and delays. In March 2016, the English National Health Service (NHS) rejected an application for a graduated roll-out of PrEP claiming that more research was needed to answer “unanswered questions.” In addition, and perhaps more critical to delay, the NHS insisted that as a prevention measure, PrEP had to be paid for by local boroughs, since prevention responsibility is held at borough-level, not nationally [9].

There are still groups that are hard to reach and do not yet feel that PrEP is a prevention intervention they have ownership over, including young women in Africa and young black gay men in the United States. PrEP is still a long way from becoming possible or even relevant in areas where the struggle for adequate treatment for people with HIV is still very much a reality. Some of the arguments about PrEP stem from deeply held beliefs that may not be easy to change. But HIV activism has always consisted of coalitions between unlikely partners such as radical gay men and church leaders, and there is no reason this cannot also be true of PrEP. There are other obstacles, including tight health budgets, stigma against sexual minorities and the inertia associated with entrenched practices of health service provision.

If PrEP is a demand-driven intervention, that demand is starting to make itself heard in various ways around the world despite opposition.
